# Effects of sweeteners on host physiology by intestinal mucosal microbiota: Example-addition sweeteners in Qiweibaizhu Powder on intestinal mucosal microbiota of mice with antibiotic-associated diarrhea

**DOI:** 10.3389/fnut.2022.1038364

**Published:** 2022-10-20

**Authors:** Bo Qiao, Jing Liu, Nenqun Xiao, Zhoujin Tan, Maijiao Peng

**Affiliations:** ^1^College of Chinese Medicine, Hunan University of Chinese Medicine, Changsha, China; ^2^College of Pharmacy, Hunan University of Chinese Medicine, Changsha, China

**Keywords:** sweeteners, intestinal mucosal microbiota, antibiotic-associated diarrhea, host physiology, Qiweibaizhu Powder

## Abstract

In recent years, sweeteners have gained massive popularity under the trend of limiting sugar intake. Our previous study found that Qiweibaizhu Powder (QWBZP) could improve gut microbiota dysbiosis and has good efficacy in treating antibiotic-associated diarrhea (AAD). In this study, we investigated the effects of sucrose, sorbitol, xylitol, and saccharin on the intestinal mucosal microbiota of AAD mice treated with QWBZP. When the AAD model was constructed by being gavaged mixed antibiotic solution, Kunming mice were randomly assigned to seven groups: the control (mn) group, the ADD (mm) group, the QWBZP (mq) group, the saccharin + QWBZP (mc) group, the sucrose + QWBZP (ms) group, the xylito + QWBZP (mx) group, and the sorbitol + QWBZP (msl) group. Subsequently, 16S rRNA gene amplicon sequencing was used to analyze the intestinal mucosal microbiota composition and abundance. The results showed that feces from AAD mice were diluted and wet and improved diarrhea symptoms with QWBZP and sorbitol. In contrast, the addition of sucrose, saccharin, and xylitol delayed the healing of diarrhea. The relative abundance of intestinal mucosal microbiota showed *Glutamicibacter*, *Robinsoniella*, and *Blautia* were characteristic bacteria of the mx group, *Candidatus Arthromitus*, and *Bacteroidales_*S24-7_group as the typical bacteria of the mn group, *Clostridium*_innocuum_group as the distinct bacteria of the mm group. *Mycoplasma* and *Bifidobacterium* as the characteristic bacteria of the ms group. Correlation analysis of typical bacterial genera with metabolic functions shows that *Blautia* negatively correlates with D-Glutamine and D-glutamate metabolism. *Bacteroidales*_S24-7_group has a significant negative correlation with the Synthesis and degradation of ketone bodies. The study confirmed that sucrose, sorbitol, xylitol, and saccharin might further influence metabolic function by altering the intestinal mucosal microbiota. Compared to the other sweetener, adding sorbitol to QWBZP was the best therapeutic effect for AAD and increased the biosynthesis and degradation activities. It provides the experimental basis for applying artificial sweeteners in traditional Chinese medicine (TCM) as a reference for further rational development and safe use of artificial sweeteners.

## Introduction

Antibiotic-associated diarrhea (AAD) is a self-limiting disease mediated by side effects and the overuse of antibiotics (1). The human gut microbiome represents a complex ecosystem contributing essential functions to its host (2). The gut microbiota interacts with the host and not only directly affects the host’s intestinal environment but also indirectly affects the host’s health by regulating the endocrine, energy metabolism, and immune function (3–5). Whereas short-term effects of antibiotics have been acknowledged in several studies, long-term consequences of the hosts‘ inhabitants remain less known but might have tremendous socioeconomic repercussions (6). The gut microbiota dysbiosis’s common features include a loss of taxonomic and functional diversity combined with reduced colonization resistance against invading pathogens, harboring the danger of antimicrobial resistance. Our previous research found alterations in the gut contents and mucosal microbiota composition of mice with AAD (7, 8). Therefore, treatment of AAD is not just about inhibiting the production of harmful bacteria but about regulating the balance of intestinal microorganisms and increasing the interaction between gut microbiota.

Qiweibaizhu Powder (QWBZP) is a classic formula for treating pediatric diarrhea, which has the effect of invigorating the spleen and producing fluid, promoting Qi, and eliminating swelling. Research has found that QWBZP has a therapeutic effect on the mucosal thickness and the number of mucosal lymphocytes in intestinal mice with AAD (9, 10). Long et al. investigated the therapeutic mechanism of QWBZP on AAD and indicated that QWBZP has a positive effect on the recovery of bacterial lactase gene diversity to normal levels (11). Although primary studies have demonstrated the efficacy of QWBZP in regulating the gut microbiota of AAD mice, its clinical application is limited due to the taste of traditional Chinese medicine (TCM). Consequently, there is an urgent to find excipients that improve effectiveness and flavors, enhance medication adherence in patients, and promote the application of TCM in the clinic.

**FIGURE 1:**
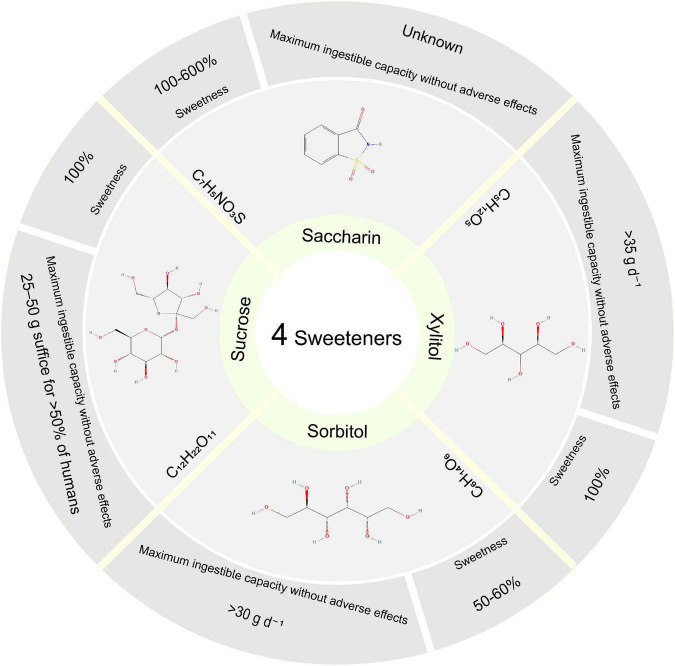
Four sweetener structures, sweetness, and maximum ingestible capacity without adverse effects. Sweetness (vs. Sucrose). Maximum ingestible capacity without Xylitol and Sorbitol’s adverse effects without sucrose’s damaging effects. Structure from PubChem Substance database.

Sweeteners, one of the most commonly used excipients, are often added to TCM to improve flavor. Although several studies have suggested that the consumption of artificial sweeteners, such as aspartame and saccharin, might have negative effects, the potential impacts of natural sweeteners on human health remain largely unknown. Our previous study found that adding sucrose to QWBZP on AAD was less effective, especially on immune function (12). Sorbitol is about half the sweetness of sucrose. Li et al. found that long-term sorbitol consumption may change the composition of the gut microbiome and potentially induce glucose intolerance (13). Xylitol is a widely used natural sweetener to reduce excessive sugar consumption. However, concerns about xylitol consumption existed as it is a highly absorbent substance in the colon that could cause diarrhea and other adverse symptoms. The metabolism of xylitol is not regulated by insulin, so it is a source of heat energy for people with diabetes (14). The intestinal microbiota has always fermented the non-digestible plant polysaccharides (15). Uebanso et al. found that xylitol altered the composition of gut microbiota and serum lipid parameters (16). So are artificial sweeteners reliable “sugar substitute” substances? Will it harm our health? In this study, we investigated the effects of sucrose, sorbitol, xylitol, and saccharin on the intestinal mucosal microbiota of AAD treated with QWBZP, which reveal the mechanism of sweetener intervention on the formula. Figure 1 briefly introduces the commonly used sweeteners: sucrose, sorbitol, xylitol, and saccharin (17).

## Materials and methods

### Animals

Forty-two Kunming mice (half of the females and males, 20 ± 2 g) were purchased from Hunan Slaccas Laboratory Animal Company (Hunan, China) with license number SCXK (Xiang) 2014-0012 according to the experimental requirements. Then the female and the male mice were given separate housings. These mice were kept in the Experimental Animal Center of Hunan University of Chinese Medicine under controlled conditions (temperature 23–25°C, humidity 47–53%). The diet compositions are corn starch 30%, soybean meal 29%, wheat 26%, salt 1%, bone meal 1%, lysine 1%, and water 12%. Animal experiments were conducted under animal protocols approved by the Animal Ethics and Welfare Committee of Hunan University of Chinese Medicine.

### Medicine

According to the Chinese Pharmacopeia 2020, QWBZP consists of *Panax ginseng* C. A. Mey., *Aucklandia lappa* Decne., *Poria cocos* (Schw.) Wolf, *Atractylodes macrocephala* Koidz., *Pueraria lobata* (Willd.) Ohwi, *Pogostemon cablin* (Blanco) Benth, *Glycyrrhiza uralensis* Fisch. All herbal medicines were purchased from the First Affiliated Hospital of Hunan University of Chinese Medicine, and the composition of the formula is shown in Table 1.

**TABLE 1:** The ingredients of QWBZP.

Chinese name	Latin name	Place	Part used	Amount (g)
Renshen	*Panax ginseng* C. A. Mey.	Shanxi	Root	6
Muxiang	*Aucklandia lappa* Decne.	Yunnan	Root	6
Fuling	*Poria cocos* (Schw.) Wolf	Yunnan	Nucleus	10
Baizhu	*Atractylodes macrocephala* Koidz.	Zhejiang	Rhizome	10
Gegen	*Pueraria lobata* (Willd.) Ohwi	Hunan	Root	10
Huoxiang	*Pogostemon cablin* (Blanco) Benth	Guangdong	Leaves	10
Gancao	*Glycyrrhiza uralensis* Fisch.	Inner Mongolia	Root	3

### Mixed antibiotic solution

A mixed antibiotic solution of gentamicin sulfate injection (Yichang Renfu Pharmaceutical Co., Ltd., State Drug Quantifier H42022058, product batch number: 5120106) and cephradine capsule (Suzhou Sinochem Pharmaceutical Industry Co., Ltd., product batch number: 110804) was prepared to make a concentration of 62.5 g/L [i.e., 6 gentamicin (2 mL/branch) + 3 cephalosporins (0.25 g/grain)] and then reserved at 4^°^C (8, 18).

### Modeling and treatment

The AAD model was constructed following the previous methods (18–20): Thirty-six mice were gavaged with an antibiotics mixture of gentamicin sulfate and cephradine (23.33 mL⋅kg^–1^⋅d^–1^) as AAD mice, six mice were gavaged with sterile water as the normal (mn) group, 0.35 mL twice daily for 5 days. When diarrheal symptoms were induced, 36 mice were randomly divided into the (mm) group, the QWBZP (mq) group, the saccharin + QWBZP (mc) group, the sucrose + QWBZP (ms) group, the xylito + QWBZP (mx) group, and the sorbitol + QWBZP (msl) group, with six mice (three males and three females) in each group.

**FIGURE 2:**
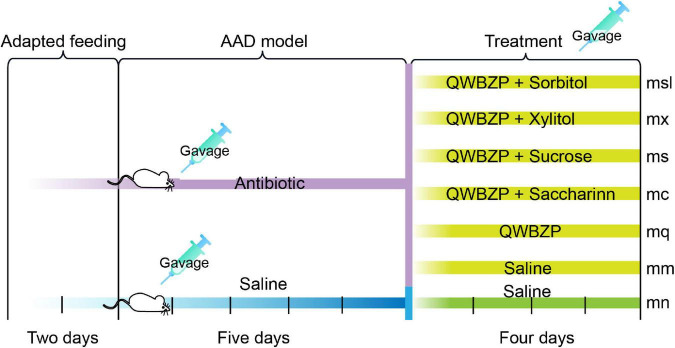
Experimental design and general conditions of the animals. mn, Control group; mm, Model group; mq, QWBZP group; ms, QWBZP + Sucrose group; mc, QWBZP + Saccharinn group; mx, QWBZP + Xylitol group; msl, QWBZP + Sorbitol group.

After the AAD model was established, mice in the mn and mm groups were gavaged with sterile water. Mice in the mq, ms, mc, mx, and msl groups were gavaged with 0.693 g⋅kg^–1^⋅d^–1^ QWBZP. In addition, the ms, mc, mx and msl were added separately 8.1 g⋅kg^–1^⋅d^–1^ sucrose, 0.169 g⋅kg^–1^⋅d^–1^ saccharin, 10 g⋅kg^–1^⋅d^–1^ xylitol, 9.52 g⋅kg^–1^⋅d^–1^ sorbitol, respectively. The gavage was administered twice daily for 4 days at a dose of 0.35 mL. The procedure is shown in Figure 2.

### Degree of wet and dry feces

The fecal contamination filter paper is divided into four grades according to the size of the stained area (21): Grade 1 is contamination diameter < 1 cm, indicated by “+”; grade 2 is the diameter of pollution < 1.0–1.9 cm, expressed as“-”; grade 3 is the diameter of contamination < 2–3, indicated by“–”; grade 4 is the diameter of contamination > 3, characterized by “—.”

### Extraction of mice intestinal mucosa

Mice were sacrificed by cervical dislocation on the 11th day, and their intestinal mucosa (the jejunum to ileum segment) was collected separately under sterile conditions. The intestine was dissected after squeezing out the contents of the chymus, and then the intestinal wall was washed with saline. The intestinal mucosa was scraped with coverslips, and two times the weight of saline was added. It was homogenized at low speed for 1 min and then centrifuged at 3,000 r/min for 10 min. The supernatant was taken for subsequent gene extraction. To control the difference induced by gender, the intestinal mucosa of one male and one female from the same group were mixed. Then, the samples were loaded into EP tubes separately and frozen at –80°C (22, 23).

### Metagenome extraction

Metagenome DNA of intestinal mucosal microorganisms was extracted according to our previous methods (18, 23) with the following processes: acetone washing, lysozyme wall breaking, proteinase K denaturation, SDS lysis, CTAB treatment, and phenol/chloroform extraction to obtain high-quality DNA. After genomic DNA extraction, the extracted genomic DNA was detected by 1% agarose gel electrophoresis.

### PCR amplification and MiSeq metagenome sequencing

The metagenomic DNA was diluted and used as a template for the V3 + V4 variable region of bacterial 16S rDNA. PCR products were detected by 2% agarose gel electrophoresis and purified by the Axygen^®^AxyPrep DNA gel extraction kit (Axygen Scientific Inc., Union City, CA, USA). And the quantified by QuantiFluorTM-ST Blue Fluorescence Quantification System (Promega), in which the corresponding proportion was mixed according to the sequencing volume requirement of each sample. A TransStart Fastpfu, DNA Polymerase 20-μL reaction system, was used, including 2.0 μL of 10 × buffer, 2.0 μL of 2.5 m MdNTPs, 0.8 μL of forwarding primer (5 μmol/L), 0.8 μL of reverse primer (5 μmol/L), 0.2 μL of Taq polymerase, 0.2 μL of BSA, 10 μL of template DNA, and 20 μL of ddH2O (23). The PCR products were then sequenced by the Illumina MiSeq sequencing platform (Illumina, San Diego, CA, USA) by Wuhan Fraser Genetic Information Co., Ltd.

### Bioinformatics

The effective sequences are classified into OTU with a similarity of 97%, and the representative OTU is classified. Chao 1 and ACE index are centered on community richness. Shannon and Simpson’s index is centered on community uniformity. Beta diversity analysis used the Bray-Curtis metric to investigate structural variation in microbial communities between samples, visualized by principal Coordinate analysis (PCoA). PCoA analysis evaluated the similarity between samples based on Euclidean distances and did not consider the possible interrelationships between the original variables. Linear discriminant analysis (LEfSe) was an analytical method based on LEfSe of effect sizes, which essentially combines LEfSe with non-parametric Kruskal-Wallis and Wilcoxon rank sum tests to screen for critical biomarkers. In addition, we classified the Kyoto Encyclopedia of Genes and Genomes using PICRUSt. Correlations between metabolic functions and characteristic bacteria were analyzed using the Person method. And the effects of different sweeteners on metabolic functions were investigated according to the degree of correlation.

### Statistical analysis

All data in the experiments were expressed as mean ± standard (SD). Statistical analysis was performed using a one-way ANOVA with LSD or rank sum test with Kruskal-Wallis for analysis among groups. The results were considered significant when *P* < 0.05. Analyzes were performed using IBM SPSS Statistics 24.0 (IBM, Corporation, Armonk, New York, NY, USA).

## Results

### Effect of sweetener on the dryness and wetness of feces in mice with antibiotic-associated diarrhea treated with Qiweibaizhu Powder

As can be seen in Table 2, the feces of all groups of mice were dilute and wet after modeling, except for the mn group. After 4 days of treatment, the degree of damp and dry feces of mice in the mq and msl groups returned to normal. The feces of the mx group were still brownish-yellow and dilute and wet. These results indicated that the intervention of QWBZP and sorbitol improved the mice with AAD, while sucrose, saccharin, and xylitol could delay the diarrhea recovery.

**TABLE 2:** Degree of dry and wet feces of mice.

Group	After modeling	After administration
mn	+	+
mm	–	–
mq	–	+
ms	–	–
mc	–	–
mx	–	–
msl	–	+

“+”, dryness of feces, “–”, degree of fecal dilution, “–” the more, the higher the dilute humidity.

### Effect of sweeteners on the structure of intestinal mucosal microbiota in mice with antibiotic-associated diarrhea treated with Qiweibaizhu Powder

**FIGURE 3:**
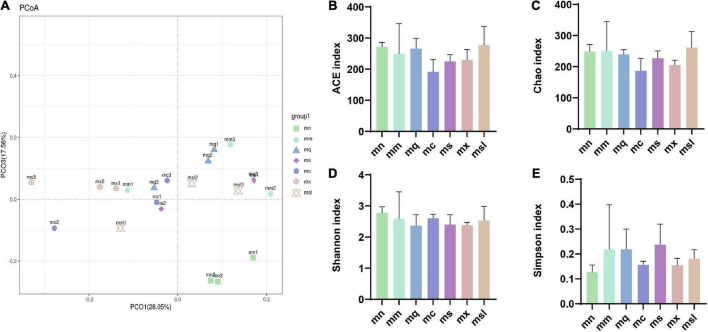
The altered bacterial diversity and richness of the intestinal mucosal microbiota in AAD mice treated with QWBZP with different sweeteners. **(A)** Principal component analysis. **(B–E)** ACE index, Chao1 index, Shannon index, Simpson index (Data were mean ± SD, One-way ANOVA with LSD or rank sum test with Kruskal-Wallis, *p < 0.05). mn, Control group; mm, Model group; mq, QWBZP group; ms, QWBZP + Sucrose group; mc, QWBZP + Saccharinn group; mx, QWBZP + Xylitol group; msl, QWBZP + Sorbitol group.

The Good’s coverage of samples exceeded 99.84%, indicating that most sequences were detected in the intestinal mucosal microbiota (356 OTUs). PCoA analyzes the similarities or differences in the composition of sample communities. Figure 3A shows that the samples of the mn group are distributed in the fourth quadrant, and the mm group is distributed in the first and second quadrants. Samples from other intervention groups are distributed in the first, second, and third quadrants. The mn group was relatively discrete from the mm group, indicating an alteration of the intestinal mucosal microbiota in mice with AAD. The above shows the difference in the overall structure of intestinal mucosal microbiota in QWBZP-treated AAD mice after adding sweetener.

Alpha diversity reflects the abundance and diversity of microbial communities, where the ACE index and Chao1 index are used to estimate the abundance of the microbiota, and the Shannon index and Simpson index reflect the diversity of the microbiota. As shown in Figure 3B, the ACE index was from high to low for the msl group, the mn group, the mq group, the mm group, the mx group, the ms group, and the mc group, and there was no significant difference between the groups (*P* > 0.05). Chao1 index was from high to low for the msl, the mm, the mn, the mq, the ms, the mx, and the mc groups, with no significant difference between the groups (*P* > 0.05) (Figure 3C). Shannon index from high to low the mn group, the mc group, the mm group, the msl group, the ms group, the mx group, and the mq group, no significant difference between the groups (*P* > 0.05) (Figure 3D), respectively. Simpson index from high to low the ms group, the mm group, the mq group, the msl group, the mc group, the mx group, and the mn group, no significant difference between the groups (*P* > 0.05) (Figure 3E), respectively. Although the differences in the richness and homogeneity of the sample microbiota among the groups were insignificant, the richness and diversity of the intestinal mucosal microbiota of AAD mice were relatively high. In contrast, the QWBZP intervention and the addition of the four sweeteners resulted in a slight decrease in intestinal mucosal microbiota richness and diversity in mice. An increase or decrease in the abundance and diversity of the microbiota does not directly indicate a good or bad gut microbiota, and the composition of the gut microbiota had to be further analyzed.

### Effect of sweeteners on the composition of intestinal mucosal microbiota in mice with antibiotic-associated diarrhea treated with Qiweibaizhu Powder

**FIGURE 4:**
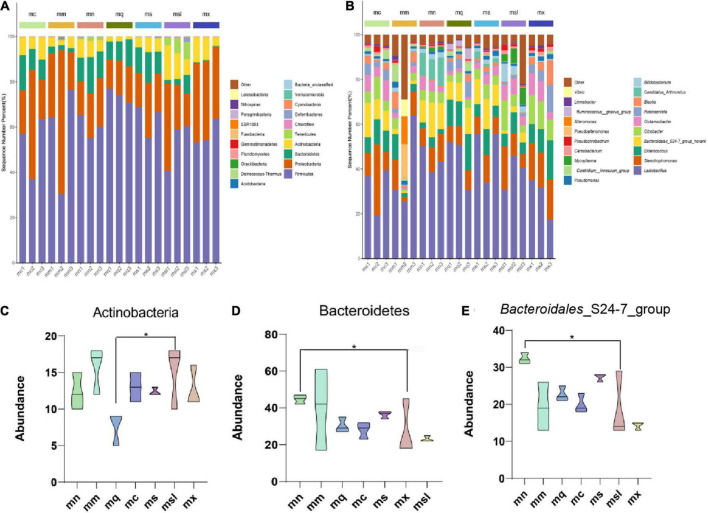
Relative abundance of the gut microbiota at the phylum and genus. **(A)** Intestinal mucosal microbiota composition in the phylum level. **(B)** Intestinal mucosal microbiota composition in the genus level. **(C–E)** Phylum and genus levels of dominant bacteria (Data were mean ± SD, One-way ANOVA with LSD or rank sum test with Kruskal-Wallis, *p < 0.05). mn, Control group; mm, Model group; mq, QWBZP group; ms, QWBZP + Sucrose group; mc, QWBZP + Saccharinn group; mx, QWBZP + Xylitol group; msl, QWBZP + Sorbitol group.

The composition of the intestinal mucosal microbiota was analyzed from different taxonomic levels. At the phylum and genus levels, there were similarities in the composition of the intestinal mucosal microbiota among the groups but some differences in relative abundance. And the highest proportions in each group were Firmicutes, Actinobacteria, Tenericutes, Proteobacteria, and Bacteroidetes (Figure 4A). Among them, Actinobacteria was increased in the msl group compared with the mq group (*P* < 0.05) (Figure 4C); Bacteroidetes was significantly decreased in the mx group than in the mn group (*P* < 0.05) (Figure 4D). The results showed that the dominant genus of the mouse was *Lactobacillus*, *Citrobacter*, *Glutamicibacter*, *Enterococcus*, *Stenotrophomonas*, *Blautia*, *Robinsoniella*, *Bifidobacterium*, *Bacteroidales*_S24-7_group (Figure 4B). *Bacteroidales*_S24-7_group belonged to Bacteroidetes, and compared with the mn group *Bacteroidales*_S24-7_group was significantly decreased in the mx group (*P* < 0.05) (Figure 4E). These results suggest that the relative abundance of intestinal mucosal microbiota in AAD mice was altered. Adding xylitol and sorbitol to QWBZP significantly affected the relative abundance of intestinal mucosal microbiota in AAD mice.

### The characteristics of intestinal mucosal microbiota of mice with the different sweeteners

**FIGURE 5:**
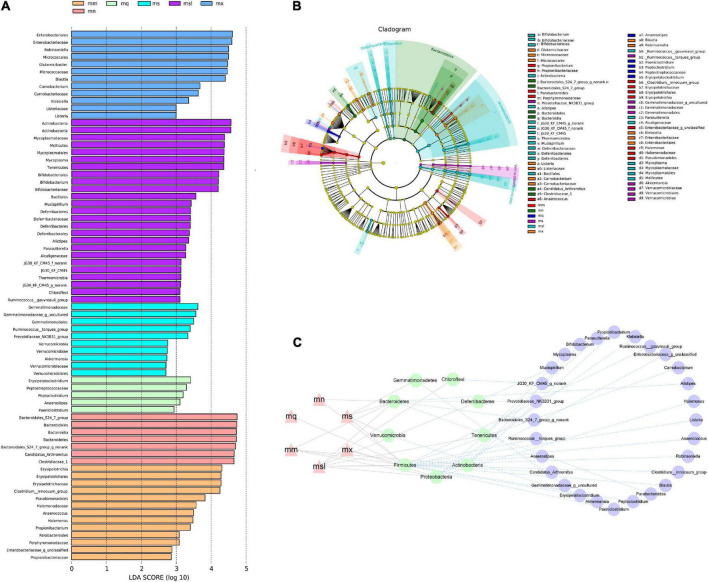
Differential bacterial taxa between different sweeteners. **(A)** Cladogram diagram. **(B)** LDA scores Chart. **(C)** Interaction network of “group—phylum—genus”. mn, Control group; mm, Model group; mq, QWBZP group; ms, QWBZP + Sucrose group; mc, QWBZP + Saccharinn group; mx, QWBZP + Xylitol group; msl, QWBZP + Sorbitol group.

Identify colonies that differed significantly between groups using LEfSe analysis (Figures 5A–C). We evaluated the top 50 genera with significant relative abundance differences, showing the typical association between bacteria at the phylum and genus. Intercomparisons between groups revealed significant differences for *Lactobacillus* in the mn group, *Anaerostipes*, *Ochrobactrum*, *Rhodobacteraceae* unclassified, *Alteromonas* in the mm group considerable difference, the mq group *Bacillus* significant difference, the ms group *Limnobacter*, *Faecalibaculum* significant difference, mx group *Stenotrophomonas*, *Bacteroidales*_S24-7_group, *Leucobacter* substantial difference. The msl group *Citrobacter*, *Glutamicibacter*, *Robinsoniella*, *Erysipelatoclostridium*, *Marinobacter*, *Lachnospiraceae* uncultured, and *Parasutterella* had significant differences.

### Effect of sweeteners on functional changes of intestinal mucosal microbiota in mice with antibiotic-associated diarrhea treated with Qiweibaizhu Powder

**FIGURE 6:**
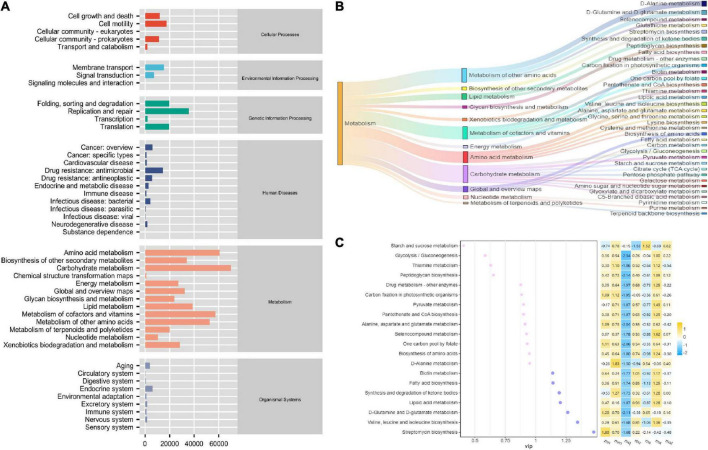
PiCRUSt-based examination of the gut microbiome of the different sweeteners. **(A)** Predictive abundance diagram of function. **(B)** Sankey diagram. **(C)** Point stick heat map.

To investigate the effect of sweeteners on the function of the intestinal mucosal microbiota in QWBZP-treated AAD mice, we analyzed the function of the intestinal mucosal microbiota using PICRUSt. The functions of the intestinal mucosal microbiota are usually divided into six major categories, with the second level including 46 functional categories and metabolic functions accounting for a greater abundance (Figure 6A). There were 152 metabolic functions, of which > 2 times the median value (> 4624.500668) had 34 functional categories (Figure 6B), and the VIP value described the overall contribution of each variable to the model, with the more significant contribution of VIP value > 1. As shown in Figure 6C, Valine, leucine, and isoleucine biosynthesis, Streptomycin biosynthesis, Fatty acid metabolism, Synthesis, and degradation of ketone bodies, and Lipoic acid metabolism have an enormous contribution. The heat map shows the changes in the expression of the variables in the variables groups, and it is seen that the metabolic function showed a significant decrease in the mq and ms groups. In conclusion, QWBZP intervention reduced the biosynthetic and degradation activity, which altered the intestinal microenvironment. The metabolic functions of the sweeteners differed due to the different characteristic bacteria.

### Correlation analysis between intestinal mucosal microbiota metabolic function

**FIGURE 7:**
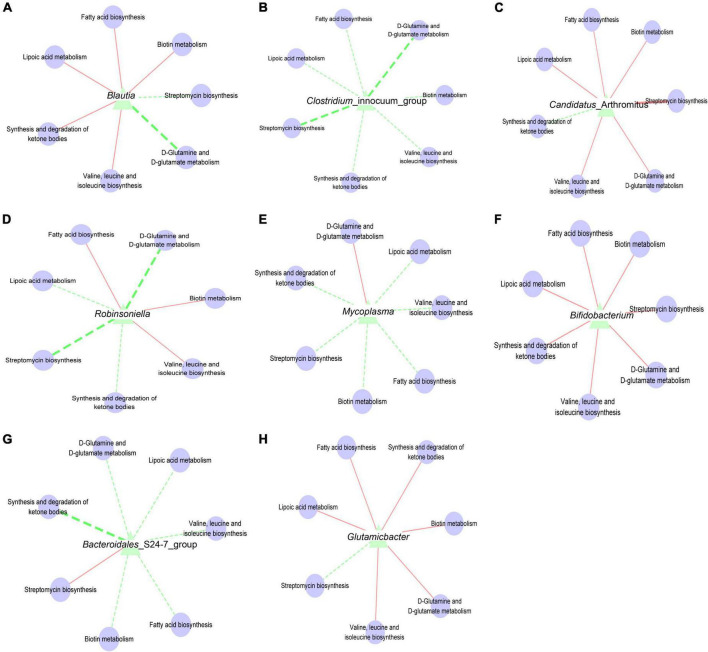
**(A)** Interaction network of “*Blautia*-metabolic function”. **(B)** Interaction network of “*Clostridium*_innocuum_group-metabolic function”. **(C)** Interaction network of “*Candidatus*_Arthromitus-metabolic function”. **(D)** Interaction network of “*Robinsoniella*-metabolic function”. **(E)** Interaction network of “*Mycoplasma*-metabolic function”. **(F)** Interaction network of “*Bifidobacterium*-metabolic function”. **(G)** Interaction network of “*Bacteroidales*_S24-7_group-metabolic function”. **(H)** Interaction network of “*Glutamicibacter*-metabolic function”. The solid line represented a positive correlation; the dotted line represented a negative correlation. The thickness of the line indicated the strength of the correlation.

In the intestinal micro-ecosystem, the gut microbiota and metabolic functions play a crucial role in maintaining the stability of the local microenvironment, but their functional relevance is unclear. Eight bacterial species (*Blautia*, characteristic bacteria of the mx group; *Candidatus_*Arthromitus and *Bacteroidales*_S24-7_group, typical bacteria of the mn group; *Clostridium_*innocuum_group, typical bacteria of the mm group; *Glutamicibacter* and *Robinsoniella*, specific bacteria of the mx group; *Mycoplasma* and *Bifidobacterium*, particular bacteria of the msl group) and seven metabolic pathways were correlated. *Blautia* is negatively associated with D-Glutamine and D-glutamate metabolism (Figure 7A). *Clostridium_*innocuum_group and *Robinsoniella* are negatively correlated with D-Glutamine and D-glutamate metabolism, Streptomycin biosynthesis (Figures 7B,D). *Candidatus_*Arthromitus is positively associated with Streptomycin biosynthesis (Figure 7C). *Bacteroidales*_S24-7_group is positively correlated with the Synthesis and degradation of ketone bodies (Figure 7G). These dynamic correlation networks reflect the functional correlation between local microbial communities and metabolites. Supplement: *Glutamicibacter* is positively correlated with D-Glutamine and D-glutamate metabolism; *Mycoplasma* is negativel associated with Streptomycin biosynthesis (Figures 7E,H).

## Discussion

The modern micro-ecological theory holds gut microbiota dysbiosis is an essential mechanism in the pathogenesis of diarrhea (24). When a large number of normal intestinal bacteria are reduced, the intestine loses the barrier and antagonistic effect of anaerobic bacteria, which is conducive to the entry of pathogenic and conditionally pathogenic bacteria, efficiently contributing to the occurrence of diarrhea (25). Modern medicine treats diarrhea mainly with antibiotics, which tend to destroy the structure of the normal microbiota, causing changes in its species and quantity. Antibiotics may lead to microbiota composition and function disorder, resulting in drug resistance genes and microecology disorders. Blind application of antibiotics in patients with diarrhea eliminates the sensitive beneficial bacteria, resulting in aggravation of dysbacteriosis (6). Gut dysbiosis promotes the horizontal transfer of resistance genes and fuels the evolution of drug-resistant pathogens and their spread. QWBZP contains flavonoids, amino acids, sterols, and other active ingredients, which are essential to inhibit harmful microbiota, promote the proliferation of beneficial microbiota and regulate the balance of microorganisms in the digestive tract (19, 26, 27). In this study, mn and mm groups’ intestinal mucosal microbial structures were different, and the intestinal mucosal microbial diversity of mm group mice showed an increasing trend. Multiple bacteria cross and reciprocate in symbiosis to form a complex system consisting of an ecosystem. Gut micro-ecological balance is controlled by antibiotics, diet, immune deficiency, or infection. Intestinal microecological dysbiosis refers mainly to a shortage of beneficial bacteria or an overgrowth of potentially harmful bacteria. Our data suggest a structural dysbiosis of the intestinal mucosal microbiota in AAD mice, and QWBZP intervention may ameliorate the dysbiosis.

Sweeteners are usually added to herbal medicines or preparations to improve the flavor of herbal medicines. The interaction of sweeteners with the intestinal microbiota affects the ecological balance of the intestinal microbiota, which impacts aspects host of metabolic health and glucose tolerance, which can affect the efficacy of herbal preparations (27, 28). This study found the symptoms of diarrhea in AAD mice improved significantly after QWBZP intervention. And the addition of sweeteners to QWBZP could delay the healing of diarrhea, mainly sucrose, saccharin, and xylose. Considering that it may be due to malabsorption, sugar alcohols accumulate in the colon and increase the colonic osmotic pressure, which may lead to gastrointestinal discomfort. However, the relationship between mal absorbed sugar alcohols, and their biological effects (e.g., osmotic diarrhea and gut microbiota composition) is currently uncertain. On the one hand, the microbiota must adapt to “unfamiliar” substrates such as sugar alcohols and artificial sweeteners. On the other, they are confronted with excessive loads of “familiar” substrates such as fructose. As a result, the microbiota composition and metabolic activities are subject to extensive modification during substrate conditioning. Evidence suggests these sugar compounds, particularly fructose, condition the microbiota, acquiring a microbiome with altered metabolic capacity.

The human gastrointestinal tract is a complex organ comprised of human and microbial genetic power, encoding many processes related to digestion, absorption, and metabolism of dietary compounds. Suez et al. demonstrate that consumption of commonly used non-caloric artificial sweeteners formulations drives the development of glucose intolerance through induction of compositional and functional alterations to the intestinal microbiota (29). Saccharin is a water-soluble acid with 85–95% excreted in the urine as a prototype. Kimmich et al. found that the action of saccharin on intestinal epithelial cells inhibited the passive transport of sugars on the side of the basement membrane and increased the intracellular glucose concentration in the intestinal cells (30). Although sucrose cannot synthesize the enzyme gene, it affects the intestinal microbiota through enzyme-like metabolism. There is a general phenomenon of inhibition in nature, where some substances with high energy use efficiency, such as sucrose, usually inhibit the use of other relatively inefficient energy substances by intestinal microor-ganisms (31). In a study published in Nature Microbiology, *Faecalibaculum* was found to exert anti-tumor effects by producing short-chain fatty acids in a mouse model of colorectal cancer (32). *Limnobacter* is a class of methanogenic bacteria that protects the environment from oxygen-sensitive anaerobic methane-oxidizing archaea (33). Jia et al. found that chlorination can effectively remove *Limnobacter* and improve antibiotic resistance (34). Neither plasma glucose nor insulin levels are affected by sugar alcohol absorption and metabolism, mainly when monosaccharide forms are consumed. Reportedly, excessive use of gum containing sorbitol causes the following symptoms diarrhea, weight loss, and bloating (35). In the present study, *Bacteroidales*_S24-7_group was significant in the mx group, and *Robinsoniella*, *Citrobacter*, and *Glutamicibacter* were substantial in the msl group. Tang et al. found that *Bacteroidales*_S24-7_group was a butyrate-producing bacterium (36). Butyrate is a major short-chain fatty acid essential for maintaining intestinal homeostasis and regulating the organ’s immune response of the organics (37). Hattori et al. found intestinal microbiota prevent sugar alcohol-induced diarrhea by degrading sorbitol in the intestine (27). *Citrobacter* is an opportunistic enterobacterium with diarrhea-causing effects (38). Therefore, it is best not to combine with sucrose when clinically using QWBZP. Therefore, when guiding the use of sweeteners, careful consideration needs to be given to the differences in sweeteners between individuals.

By predicting the metabolic function of the intestinal mucosal microbiota, we found that Streptomycin biosynthesis, Fatty acid metabolism, Synthesis and degradation of ketone bodies, Biotin metabolism, and Lipoic acid metabolism have the most significant contribution. Lipoic acid is an essential cofactor for mitochondrial metabolism and is synthesized *de novo* using intermediates from mitochondrial fatty-acid synthesis type II, S-adenosylmethionine, and iron-sulfur clusters (39). Lipoic acid plays a critical role in stabilizing and regulating these multienzyme complexes. The liver shows net glutamine uptake after a protein-containing meal, during uncontrolled diabetes, sepsis, and short-term starvation, but changes to net release during long-term starvation and metabolic acidosis. The differential expression of glutamine synthetase (perivenous) and glutaminase (periportal) within the liver indicates that glutamine is used for urea synthesis in periportal cells. In contrast, glutamine synthesis serves to detoxify any residual ammonia in perivenous cells (40). Ketosomes play an essential role in organismal energy homeostasis, mainly as oxidizing fuel, potential redox regulators, lipogenic precursors, and signaling when carbohydrates are in short supply (41). In conclusion, the alteration of intestinal mucosal microbiota function after the intervention of QWBZP with sweetener affected the intestinal microenvironment.

The present study demonstrated that AAD mice showed disorders in the structure and function of the intestinal mucosal microbiota, and QWBZP had better efficacy in the treatment of AAD, which was consistent with the results of the previous study. To improve the flavor of TCM, we added sucrose, saccharin, xylitol, and sorbitol to QWBZP. We found that different sweetener characteristic bacteria, such as *Faecalibaculum*, *Limnobacter* could be used as characteristic bacteria to distinguish sucrose from other sweeteners. *Robinsoniella*, *Citrobacter*, and *Glutamicibacter* could be used as typical bacteria to determine the sorbitol group from other sweeteners. And adding sucrose, saccharin, xylitol, and sorbitol may lead to changes in Streptomycin biosynthesis, Fatty acid metabolism, Synthesis, and degradation of ketone bodies, Biotin metabolism, and Lipoic acid metabolism, which in turn may affect the health of the organism. Our study has some shortcomings. Firstly the exploration of the dose of sweeteners used. Secondly, the intestinal microbiota is probably the biological basis of physiological and pathological differences caused by gender, and intestinal mucosa-associated microbiota can better represent the sex dimorphism of mice (42). Therefore, the influence of gender on the test results is also noteworthy. Finally, intestinal mucosal microbiota characterization and corresponding metabolites still require studies with larger sample sizes to be validated. The impact of sweetener consumption on human health still needs more studies.

## Data availability statement

The datasets presented in this study can be found in online repositories. The names of the repository/repositories and accession number(s) can be found below: https://www.ncbi.nlm.nih.gov/, PRJNA881802.

## Ethics statement

The animal study was reviewed and approved by the Animal Ethics and Welfare Committee of Hunan University of Chinese Medicine. Written informed consent was obtained from the owners for the participation of their animals in this study.

## Author contributions

BQ analyzed the data and drafted the manuscript. JL performed most of the experiments and statistical analysis. ZT and NX guided the performance of the animal experiment. MP was responsible for studying the design and collecting funds. All authors reviewed and approved the final manuscript.

## References

[1] Guo H Yu L Tian F Zhao J Zhang H Chen W Effects of *Bacteroides*-based microecologics against antibiotic-associated diarrhea in mice. *Microorganisms.* (2021) 9:2492. 10.3390/microorganisms9122492 34946094 PMC8705046

[2] Heintz-Buschart A Wilmes P. Human gut microbiome: function matters. *Trends Microbiol.* (2018) 26:563–74. 10.1016/j.tim.2017.11.002 29173869

[3] Adak A Khan MR. An insight into gut microbiota and its functionalities. *Cell Mol Life Sci.* (2019) 76:473–93. 10.1007/s00018-018-2943-4 30317530 PMC11105460

[4] Kc D Sumner R Lippmann S. Gut microbiota and health. *Postgrad Med.* (2020) 132:274. 10.1080/00325481.2019.1662711 31566046

[5] Yang J Yang Y Ishii M Nagata M Aw W Obana N Does the gut microbiota modulate host physiology through polymicrobial biofilms? *Microbes Environ.* (2020) 35:ME20037. 10.1264/jsme2.ME20037 32624527 PMC7511787

[6] Lange K Buerger M Stallmach A Bruns T. Effects of antibiotics on gut microbiota. *Dig Dis.* (2016) 34:260–8. 10.1159/000443360 27028893

[7] Xie G Tan K Peng M Long C Li D Tan Z. Bacterial diversity in intestinal mucosa of antibiotic-associated diarrhea mice. *3 Biotech.* (2019) 9:444. 10.1007/s13205-019-1967-2 31763122 PMC6842370

[8] Shao H Zhang C Xiao N Tan Z. Gut microbiota characteristics in mice with antibiotic-associated diarrhea. *BMC Microbiol.* (2020) 20:313. 10.1186/s12866-020-01999-x 33059603 PMC7559773

[9] Liu Y Li D Liu Q Hui H. Effect of *Qiwei Baizhu* powder on the intestinal mucosa of mice with dysbacteria diarrhea. *Chin J Microecol.* (2018) 30:777–80. 10.13381/j.cnki.cjm.201807007

[10] Guo K Peng X Mao Y Xu S Yang Z Tan Z. Effect of *Qiwei Baizhu* San onintestinal sucrase activity in mice with diarrhea. *Chin J Microecol.* (2019) 31:1130–4. 10.13381/j.cnki.cjm.201910003

[11] Long C Liu Y He L Yu R Li D Tan Z Bacterial lactase genes diversity in intestinal mucosa of dysbacterial diarrhea mice treated with *Qiweibaizhu* powder. *3 Biotech.* (2018) 8:423. 10.1007/s13205-018-1460-3 30280074 PMC6160371

[12] Xiao X Liu Y Deng Y Guo K Yuan Z Tan Z. The Effect of sucrose on blood routine of dysbacteriosis diarrheal mice treated with *Qiweibaizhu* powder. *J Jiangxi Univ TCM.* (2019) 42:94–8. 10.13424/j.cnki.jsctcm.2019.04.024

[13] Li C Wang C Lin Y Kuo H Wu J Hong T Long-term consumption of the sugar substitute sorbitol alters gut microbiome and induces glucose intolerance in mice. *Life Sci.* (2022) 305:120770. 10.1016/j.lfs.2022.120770 35792179

[14] Granström TB Izumori K Leisola M. A rare sugar xylitol. Part I: the biochemistry and biosynthesis of xylitol. *Appl Microbiol Biotechnol.* (2007) 74:277–81. 10.1007/s00253-006-0761-3 17216457

[15] Ji X Hou C Gao Y Xue Y Yan Y Guo X. Metagenomic analysis of gut microbiota modulatory effects of jujube (*Ziziphus jujuba Mill*.) polysaccharides in a colorectal cancer mouse model. *Food Funct.* (2020) 11:163–73. 10.1039/c9fo02171j 31830158

[16] Uebanso T Kano S Yoshimoto A Naito C Shimohata T Mawatari K Effects of consuming xylitol on gut microbiota and lipid metabolism in mice. *Nutrients.* (2017) 9:756. 10.3390/nu9070756 28708089 PMC5537870

[17] Suez J Korem T Zilberman-Schapira G Segal E Elinav E. Non-caloric artificial sweeteners and the microbiome: findings and challenges. *Gut Microbes.* (2015) 6:149–55. 10.1080/19490976.2015.1017700 25831243 PMC4615743

[18] Li C Zhou K Xiao N Peng M Tan Z. The effect of *Qiweibaizhu* powder crude polysaccharide on antibiotic-associated diarrhea mice is associated with restoring intestinal mucosal bacteria. *Front Nutr.* (2022) 9:952647. 10.3389/fnut.2022.952647 35873450 PMC9305308

[19] Hui H Wu Y Zheng T Zhou S Tan Z. Bacterial characteristics in intestinal contents of antibiotic-associated diarrhea mice treated with Qiweibaizhu powder. *Med Sci Monit.* (2020) 26:e921771. 10.12659/MSM.921771 32398636 PMC7245059

[20] Zhang CY Peng XX Shao HQ Li XY Wu Y Tan ZJ. Gut microbiota comparison between intestinal contents and mucosa in mice with repeated stress-related diarrhea provides novel insight. *Front Microbiol.* (2021) 12:626691. 10.3389/fmicb.2021.626691 33708183 PMC7940357

[21] Su X Tang Y Zhang J Bai Y Shi H Liu Y Shen warming Pi strengthening method intervened IBS-D rats: an efficacy assessment. *Zhongguo Zhong Xi Yi Jie He Za Zhi.* (2014) 34:197–202. 24672945

[22] Li X Peng X Guo K Tan Z. Bacterial diversity in intestinal mucosa of mice fed with *Dendrobium* officinale and high-fat diet. *3 Biotech.* (2021) 11:22. 10.1007/s13205-020-02558-x 33442520 PMC7779387

[23] Long CX Shao HQ Luo CY Yu R Tan ZJ. Bacterial diversity in the intestinal mucosa of *Dysbiosis* diarrhea mice treated with *Qiweibaizhu* powder. *Gastroenterol Res Pract.* (2020) 2020:9420129. 10.1155/2020/9420129 32256567 PMC7097775

[24] Li Y Xia S Jiang X Feng C Gong S Ma J Gut microbiota and diarrhea: an updated review. *Front Cell Infect Microbiol.* (2021) 11:625210. 10.3389/fcimb.2021.625210 33937093 PMC8082445

[25] Pimentel M Lembo A. Microbiome and its role in irritable bowel syndrome. *Dig Dis Sci.* (2020) 65:829–39. 10.1007/s10620-020-06109-5 32026278

[26] Xie G Deng N Zheng T Peng X Zhang S Tan Z. Total glycosides contribute to the anti-diarrheal effects of *Qiwei Baizhu* powder via regulating gut microbiota and bile acids. *Front Cell Infect Microbiol.* (2022) 12:945263. 10.3389/fcimb.2022.945263 36072221 PMC9444044

[27] Hattori K Akiyama M Seki N Yakabe K Hase K Kim YG. Gut microbiota prevents sugar alcohol-induced diarrhea. *Nutrients.* (2021) 13:2029. 10.3390/nu13062029 34204751 PMC8231616

[28] Daly K Darby AC Shirazi-Beechey SP. Low calorie sweeteners and gut microbiota. *Physiol Behav.* (2016) 164:494–500. 10.1016/j.physbeh.2016.03.014 26992958

[29] Suez J Korem T Zeevi D Zilberman-Schapira G Thaiss CA Maza O Artificial sweeteners induce glucose intolerance by altering the gut microbiota. *Nature.* (2014) 514:181–6. 10.1038/nature13793 25231862

[30] Kimmich GA Randles J Anderson RL. Inhibition of the serosal sugar carrier in isolated intestinal epithelial cells by saccharin. *Food Chem Toxicol.* (1988) 26:927–34. 10.1016/0278-6915(88)90091-9 3209132

[31] Ahmad SY Friel J Mackay D. The effects of non-nutritive artificial sweeteners, aspartame and sucralose, on the gut microbiome in healthy adults: secondary outcomes of a randomized double-blinded crossover clinical trial. *Nutrients.* (2020) 12:3408. 10.3390/nu12113408 33171964 PMC7694690

[32] Zagato E Pozzi C Bertocchi A Schioppa T Saccheri F Guglietta S Endogenous murine microbiota member *Faecalibaculum rodentium* and its human homologue protect from intestinal tumour growth. *Nat Microbiol.* (2020) 5:511–24. 10.1038/s41564-019-0649-5 31988379 PMC7048616

[33] Chen Y Feng X He Y Wang F. Genome analysis of a *Limnobacter sp*. identified in an anaerobic methane-consuming cell consortium. *Front Mar Sci.* (2016) 3:257. 10.3389/fmars.2016.00257

[34] Jia S Shi P Hu Q Li B Zhang T Zhang XX. Bacterial community shift drives antibiotic resistance promotion during drinking water chlorination. *Environ Sci Technol.* (2015) 49:12271–9. 10.1021/acs.est.5b0352 26397118

[35] Corazza GR Strocchi A Rossi R Sirola D Gasbarrini G. Sorbitol malabsorption in normal volunteers and in patients with coeliac disease. *Gut.* (1988) 29:44–8. 10.1136/gut.29.1.44 3343011 PMC1433267

[36] Tang W Yao X Xia F Yang M Chen Z Zhou B Modulation of the gut microbiota in rats by Hugan Qingzhi tablets during the treatment of high-fat-diet-induced nonalcoholic fatty liver disease. *Oxid Med Cell Longev.* (2018) 2018:7261619. 10.1155/2018/7261619 30671174 PMC6323444

[37] Serino M. SCFAs - the thin microbial metabolic line between good and bad. *Nat Rev Endocrinol.* (2019) 15:318–9. 10.1038/s41574-019-0205-7 30976118

[38] Oyeka M Antony S. *Citrobacter braakii* bacteremia: case report and review of the literature. *Infect Disord Drug Targets.* (2017) 17:59–63. 10.2174/1871526516666161005155847 27658860

[39] Solmonson A DeBerardinis RJ. Lipoic acid metabolism and mitochondrial redox regulation. *J Biol Chem.* (2017) 293:7522–30. 10.1074/jbc.TM117.000259 29191830 PMC5961061

[40] Svenneby G Torgner IA. Localization and function of glutamine synthetase and glutaminase. *Biochem Soc Trans.* (1987) 15:213–5. 10.1042/bst0150213 2884149

[41] Fukao T Lopaschuk GD Mitchell GA. Pathways and control of ketone body metabolism: on the fringe of lipid biochemistry. *Prostaglandins Leukot Essent Fatty Acids.* (2004) 70:243–51. 10.1016/j.plefa.2003.11.001 14769483

[42] He L Liu Y Guo Y Shen K Hui H Tan Z. Diversity of intestinal bacterial lactase gene in antibiotics-induced diarrhea mice treated with Chinese herbs compound Qi Wei Bai Zhu San. *3 Biotech.* (2018) 8:4. 10.1007/s13205-017-1024-y 29242764 PMC5718989

